# A retrospective analysis on the diagnostic value of ultrasound-guided percutaneous biopsy for peritoneal lesions

**DOI:** 10.1186/1477-7819-11-251

**Published:** 2013-10-02

**Authors:** Jianhong Wang, Liucun Gao, Shanhong Tang, Tao Li, Yiming Lei, Huahong Xie, Jie Liang, Baojun Chen, Xian Wang, Daiming Fan

**Affiliations:** 1 State Key Laboratory of Cancer Biology, Xijing Hospital of Digestive Diseases, Fourth Military Medical University, Xi’an 710032, China; 2 Department of Pharmacology and Toxicology, Beijing Institute of Radiation Medicine, Beijing 100850, PR China; 3 Department of Digestion, General Hospital of Chengdu Military Command, Chengdu 610083, Sichuan Province, China

**Keywords:** Ultrasonography, Guidance, Biopsy, Peritoneal lesions, Omental lesions

## Abstract

**Background:**

Routine examinations have a low specificity and a low positive rate for the diagnosis of peritoneal lesions. This study aimed to evaluate the diagnostic value and safety of ultrasound-guided percutaneous peritoneal lesion biopsies in patients with ascites and/or abdominal distension with unclear causes.

**Methods:**

A retrospective analysis was performed in 153 consecutive patients with ascites and/or abdominal distension with unclear causes. All of the patients showed abnormalities of the peritoneum or greater omentum after ultrasonography, and underwent ultrasound-guided percutaneous biopsies using a Bard auto-biopsy gun with 18- or 16**-**gauge biopsy needles.

**Results:**

The success rate of the procedures was 100% (153/153) and the satisfaction rate of the tissue specimens in the biopsy was 91.5% (140/153). A specific histopathological diagnosis was made in 142 out of 153 patients, with an overall diagnostic accuracy of 92.8%. Among the diagnosed patients, 62 were peritoneal metastatic adenocarcinoma, 49 were peritoneal tuberculosis, 11 were peritoneal malignant mesothelioma, 8 were chronic peritoneal infections, 7 were pseudomyxoma peritonei, and 5 were primary peritoneal lymphoma. Only 11 patients did not get a pathologic diagnosis due to the lack of sufficient tissue specimen. No serious complications occurred.

**Conclusions:**

Ultrasound-guided percutaneous biopsy could be a simple, safe and accurate diagnostic method in patients with ascites and/or abdominal distension with unclear causes.

## Background

Peritoneal diseases are present in many patients with ascites and/or abdominal distention with unclear reasons. In northwest China, peritoneal tuberculosis is a common disease, yet it remains a big challenge for doctors to make an accurate diagnosis. The conventional examinations have a low specificity and a low accuracy for the diagnosis of peritoneal lesions, making it hard to identify its etiopathogenesis, and subsequently give clinical doctors many difficulties in treating this disorder. Up to the present, only a few reports on patients with peritoneal lesions undergoing imaging-guided percutaneous biopsy have been published [1-5], probably due to the difficulty of finding the peritoneal disorder in the conventional imaging method for peritoneal diseases. Computed tomography (CT)-guided percutaneous biopsy is not a real-time operation, and it involves quite a few complicated procedures. Laparoscopy can detect peritoneal lesions and provide biopsies for the different parts of these lesions while maintaining high diagnostic accuracy. However, laparoscopy involves complex manipulations with many complications and requires anesthesia in an operating room, thus introducing risk for the patient. For these reasons, laparoscopy is still not a very popular choice in this situation.

Ultrasonography is an ideal method for imaging and for guiding a biopsy. An ultrasound-guided percutaneous biopsy is already a common method for the histodiagnosis of abdominal lesions, such as those in the liver, kidney, pancreas and other solid organs [6-8], but so far, it is not often used for peritoneal and omental lesions [9-11]. Thus, the purpose of our study is to evaluate the clinical diagnostic value and safety of ultrasound-guided percutaneous biopsies for peritoneal lesions to further analyze the etiology of these lesions.

## Methods

A total of 88 male and 65 female patients (age 11 to 75 years, average age 45.3 ± 15.6 years) with ascites and/or abdominal distention of unclear causes were included in this study. Ultrasonography showed abnormalities of the peritoneum and/or greater omentum in all cases. All patients signed informed consent forms, and the Hospital’s Protection of Human Subjects Committee approved the experiment’s protocol.

All patients underwent an ultrasound-guided percutaneous biopsy. Two ultrasonography systems (ATL Ultrasound 22100: Advanced Technology Laboratories, Inc.; IU22:Philips Ultrasound,Inc. Washington,USA) and 2–5 MHz convex array transducers were used. Bard auto-biopsy guns with 18- or 16**-**gauge biopsy needles (Bard Inc., Covington, GA, USA) were used for the biopsy. The sampling length could be adjusted to 15 or 22 mm.

Before the biopsy, the B-mode ultrasonography was performed to show the thickness, the location and the echoes of the peritoneum or omentum lesions. The color Doppler flow imaging (CDFI) was also performed to observe the vascularity in the peritoneal lesions and to determine whether there are important organs or large vessels nearby. The operation protocols were then created with the positions, angles, and depth of the punctures as well as the dangerous zones accurately pinpointed so as to make sure the shortest and safest puncture route is taken during the procedure.

Before the biopsy, the occurrence of indications was confirmed for each patient. For those with greater amounts of ascites, biopsies were not performed until ascites decreased through drainage or after treatment with diuretic agents. Prior to the biopsy, the details of this procedure were well explained to each patient and his/her family to reduce their anxiety.

The procedure was performed by two ultrasonography doctors, one holding the transducer steady and the other performing the operation. During the conventional ultrasonography, the doctor would first identify the thickest biopsy lesion and the regions of more obvious abnormal echoes or comparatively more blood flow. Then, the puncture angle and length could be adjusted to meet the optimal parameters. In addition, a local anesthetic had to be administered subcutaneously with a 25-gauge needle by infiltrating the abdominal wall (2% lidocaine hydrochloride). An incision was made in the skin, and a biopsy needle was placed straight into the deep layer of the abdominal wall. The patient was asked to hold his/her breath for a second, and during that point the biopsy needle was inserted deeper into the surface of the lesion, and then quickly withdrawn after the biopsy gun was triggered. Two samples were taken from each patient, and sometimes three or even more when it was necessary. The samples were fixed in formalin and sent to the pathological department after their colors and lengths were recorded. After the procedure, the patient was ordered to stay in bed for at least 12 h with intensive care, while their vital signs and peritoneal symptoms were monitored; very often hemostasis and anti-infective therapies were necessary.

Hematoxylin and eosin (HE) staining and microscopic observations were performed for the pathological examinations. Immunohistochemical and electron microscopy studies were performed when necessary.

## Results

### Ultrasonography

**Figure 1:**
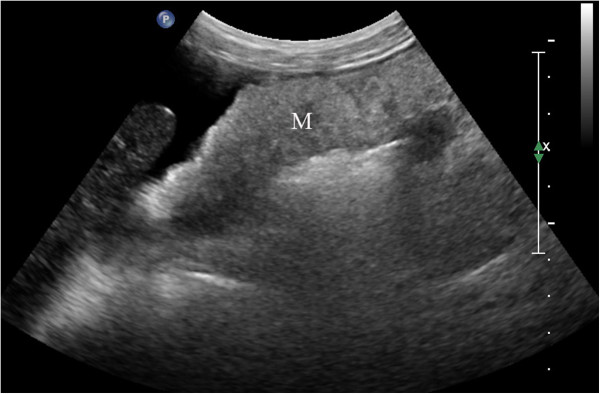
**Data for a 56-year-old woman.** Ultrasonography showed a cake-shaped thickening of the omentum and a nodosity-shaped thickening of the peritoneum (M); these thickenings were pathologically diagnosed as metastatic mucinous adenocarcinoma.

The accurate identification of the peritoneal and omental lesions by ultrasonography is essential for the success of the biopsy. Here, all of the patients had a locally or diffusely thickening peritoneum (thickness range, 0.8 to 3.7 cm; mean thickness, 1.7 ± 0.8 cm). Among all the patients, 114 had a cake-shaped thickening of the peritoneum or omentum (Figure 1), and 39 had a nodous peritoneum or omentum. Overall, 74 patients with a thickened omentum had a heterogeneous hypoechoic pattern, 59 patients had a hyperechoic pattern, and 20 patients had both patterns. The thickened peritoneum or omentum tended to be stiff, and it made the lesions hard to deform with the transducer compression. Ascites were present in 123 patients; of these, 30 had a large volume, 42 had a moderate volume, and 51 had a small volume.

### Biopsy

**Figure 2:**
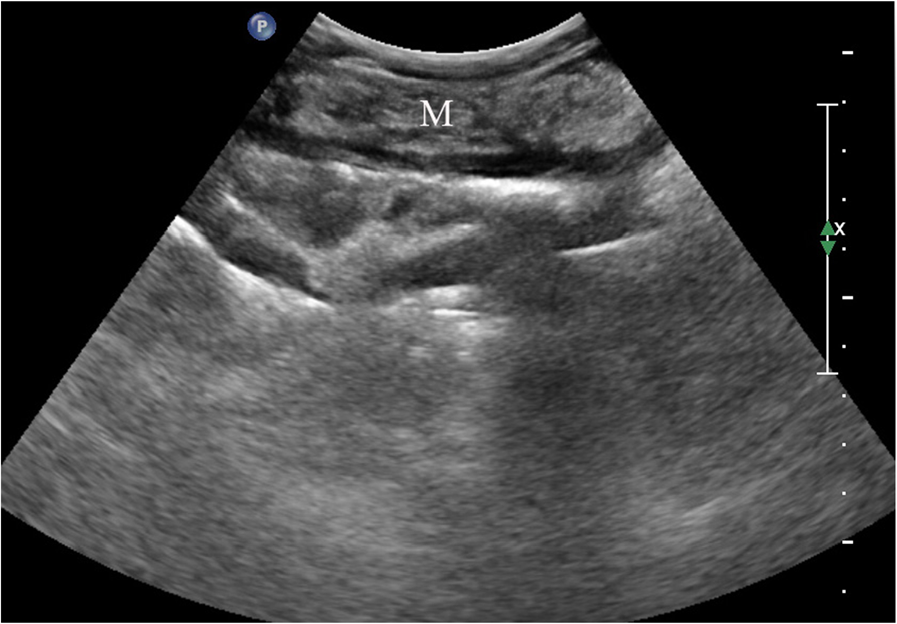
**Data for a 37-year-old man.** Ultrasonography showed a cake-shaped thickening of the omentum (M) with homogeneous hyperechoes; this thickening was then pathologically diagnosed as tuberculosis of the peritoneum.

**Figure 3:**
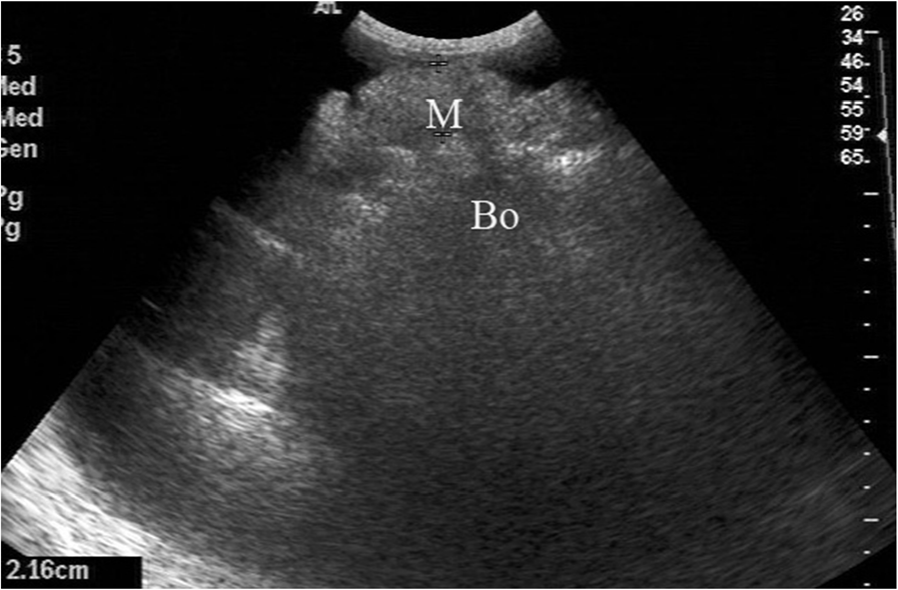
**Data for a 46-year-old woman.** Ultrasonography showed a thickening of the visceral peritoneum (M) with homogeneous hyperechoes; this thickening was pathologically diagnosed as malignant peritoneal mesothelioma.

**Figure 4:**
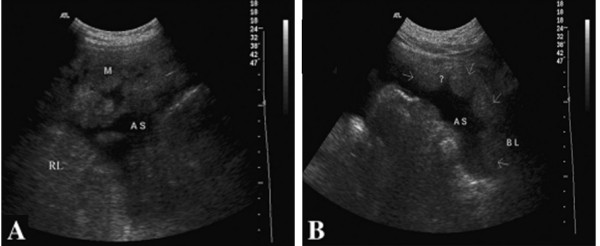
**A 44-year-old man was hospitalized with abdominal distension.** Ultrasonography showed cake- or nodosity-shaped thickening of the omentum **(A)** and peritoneum **(B)** with homogeneous hypoechoes. His pathological diagnosis was NHLL(Non-Hodgkin's lymphoma).

Biopsies were performed in 153 patients, with two to five samples taken from each patient (mean number: 2.3 ± 1.1). The success rate of the biopsies was 100% (153/153), and the satisfaction rate of the tissue specimens was 91.5% (140/153). Among the 142 cases that had a histopathologic diagnosis following the biopsy, 62 were peritoneal metastatic adenocarcinoma (Figure 1), 49 were peritoneal tuberculosis (Figure 2), 11 were peritoneal malignant mesothelioma (Figure 3), 8 were chronic peritoneal infections, 7 were pseudomyxoma peritonei, and 5 were primary peritoneal lymphoma (Figure 4). The biopsies of 13 patients failed to provide sufficient samples, and 11 patients among them were unable to obtain a pathological diagnosis. Overall, the diagnostic accuracy was 92.8% (142/153).

The proper use of a biopsy is the key point for obtaining a good sample. We concluded that a suitable increase of the puncturing angle, a longer sampling length, and the use of a coarser biopsy needle improved the satisfaction rate of the samples and the accuracy of the histopathological diagnosis. In this study, the peritoneal lesions of 11 patients were too thin, as the lesions’ thicknesses were smaller than 1 cm. These small lesions were prone to injure the intestine when biopsied using routine methods. However, by increasing the biopsy-punctured angle, these biopsies could be successfully performed, with each sample being longer than 1 cm. Meanwhile, the 16G biopsy-cut needle provided more tissue from thicker lesions. This method decreased the number of punctures without increasing the complications.

### Complications

The complications were evaluated within 12 hours after the biopsy. Fifteen patients experienced pain at the operation site, which eased off later without treatment. During ultrasonography, two cases, which showed a high-level echo flow into the abdominal cavity, were found to have bled after the biopsy; in both cases, the bleeding stopped when the transducer pressure was applied. No other serious complications occurred.

## Discussion

The main functions of peritoneum are to protect the abdominal organs and to limit inflammatory diffusion. At the same time, the peritoneum can be infected easily through inflammation, tumors and other diseases of the abdominal organs at an early stage. In the past, diagnosis of peritoneal lesions usually depended on abdominal surgery. Currently, laparoscopy can detect peritoneal lesions and allow biopsies to be obtained from various parts of peritoneal lesions. However, this procedure involves complex manipulations, and may cause little wounds, complications and may even be dangerous to perform in some cases. These drawbacks prevent laparoscopy from being popular. Patients with peritoneal or omental lesions who underwent imaging-guided percutaneous biopsies are rarely reported [9-12]. Most of the diagnoses are based on fine needle aspiration cytology, which has a low diagnostic accuracy. Only few diagnoses are based on the histopathological diagnosis in a small sample size. In contrast, ultrasound-guided percutaneous biopsies are easy to perform in an outpatient clinic. This procedure is safe, has a low incidence of injury and does not cause serious complications; it also has a high diagnostic accuracy, which makes it widely used in clinics [1,2]. In this study, we used the ultrasound-guided percutaneous biopsy to perform cut-needle biopsies of peritoneal lesions using a coarse biopsy needle and obtained good results.

Peritoneal and omental lesions can be accurately imaged by ultrasonography, which is essential for the biopsy. Ultrasonography can make any abnormality in peritoneum and omentum easily visible. Reports on the ultrasonographic diagnosis of peritoneal and omental lesions are rare because of the difficulties in making differential diagnosis of these lesions from other abdominal organs, such as the intestines. Then, how can peritoneal lesions be detected by ultrasonography? In our opinion, lesions in the visceral peritoneum are always located on the surfaces of the abdominal organs, such as the intestines and liver, and at the periphery of the gastric antrum. They have comparatively fixed locations with clear boundaries between the nearby organs. Lesions in the parietal peritoneum are always located on the inside of the abdominal wall, especially on the bottom of the pelvic cavity, the anterior wall of the abdomen and the bilateral wall of the abdominal cavity. Omental lesions have fixed locations on the anterior surface of the small intestines. They are easily identified because they have a specific thickness and hardness, are free at the inferior or bilateral extremities, and there are no enterokinesia and hyperechoic patterns, such as gas in these areas. This study showed that although the ultrasonographic images of benign and malignant lesions are different, their images could overlap, which makes the lesions hard to differentiate. For this reason, a biopsy is needed.

The key to success in a biopsy is to obtain many tissue samples while reducing complications as much as possible. Before these objectives could be achieved, it should first be confirmed that the performance of a biopsy is appropriate for the patient. In particular, in those patients with a large number of ascites, biopsy should not be performed until the ascites are reduced to the largest extent possible. Otherwise, hemostasis during the biopsy would be difficult to achieve. Secondly, selection of biopsy pathway is also crucial. Lesions that are among the thickest, that had obvious abnormal internal echoes, or that had a comparatively greater blood flow in the CDFI were selected for biopsy. In order to get more tissue samples, the needle punctured angle and sample length were adjusted to the best parameters. Third, the doctor performing biopsy should be skilled and cautious during the operation. He should clearly and decisively order the patient to hold his/her breath, and his puncture actions should be facile and dexterous to avoid injuring the lesion with the needle tip, which can result in bleeding. Fourth, the length and integrity of the samples are closely related to the accuracy of the pathological diagnosis. During the biopsy, the sampling length is always adjusted to 22 mm to ensure that sufficient samples and the maximum tissue cutting strength are available. If the lesion is thinner, the needle-punctured angle should be changed to obtain more samples. Fifth, the position of the needle tip and the pathway of the biopsy should be displayed clearly. The biopsy should never be performed blindly; otherwise, adjacent organs and tissues may be injured. Finally, the peritoneum and omentum lesions are always thickened, hardened and have a large area with a cake-like shape. These lesions are easy to fix during a biopsy. If there are any lesions moving or floating, the peritoneum and omentum should be kept steady through compression by an assistant.

Safety has been regarded as a key value of ultrasound-guided percutaneous biopsies, and many studies have already proven that this method is one of the safest to obtain a histopathological diagnosis [1-5]. Theoretically, the anatomical features of the peritoneum and omentum show that most of the lesions are superficial, adhering to the abdominal wall and thickening when the lesion occurs. Therefore, during the operation, the important organs underneath can be untouched. Furthermore, the entire biopsy process can be monitored by ultrasonography, ensuring that this procedure is comparably safe. Even if complications like bleeding occur, the ultrasound transducer can stop the bleeding with continuous pressure. Hence, ultrasound-guided percutaneous biopsies in peritoneal and omental lesions cause fewer complications than other biopsy methods do.

In this study, of the 153 patients, 142 (92.8%) received a specific histopathologic diagnosis using ultrasonographic guidance. However, a previous study using CT-guided image-guided core biopsy showed that 15 of 19 patients (79%) with omental lesions received a definite diagnosis, whereas only three of six (50%) patients eventually received benign diagnoses [12], indicating that ultrasonographic guidance may be more successful and convenient in the percutaneous biopsy in peritoneal and omental lesions. Among the peritoneal and omental lesions in this study imaged by ultrasonography, the incidence of peritoneal metastatic adenocarcinoma was the highest (43.7%, 62/142), with a value slightly larger than that of a previous report (35.7%, 65/182), and seven patients (4.9%, 7/142) had pseudomyxoma peritonei, which is also a higher incidence than in a previous report (0.23%, 1/182) for ultrasound-guided biopsies of the greater omentum. However, the incidence of primary peritoneal tumors was very low [5]. In addition, peritoneal tuberculosis is a common disease, which remains a significant diagnostic challenge for doctors in northwest China, where the incidence of peritoneal tuberculosis (34.5%, 49/142) is the second highest. In a previous study, Vardareli *et al*. reported the diagnostic efficiency for 19 patients with peritoneal tuberculosis using image-guided peritoneal biopsy; ultrasound guidance was used in 11 patients, and computed tomography (CT) guidance was used in 8. The histological examination succeeded in 18 patients, while the one remaining patient required laparoscopy for the peritoneal biopsy [13].

Our results also showed the overlapping appearance of several common peritoneal lesions in ultrasonography. Therefore, ultrasound-guided percutaneous biopsy is preferred for an accurate diagnosis of peritoneal and omental lesions once these lesions are detected.

## Conclusions

In conclusion, for patients with ascites and/or abdominal distension of unclear causes, after the confirmation of abnormalities in the peritoneum or omentum by ultrasonography or CT, an ultrasound-guided percutaneous biopsy can be used to obtain an accurate pathological diagnosis. Ultrasound-guided percutaneous biopsy is a convenient, safe and effective method with a high diagnostic accuracy. This technique offers remarkable assistance during the selection of the appropriate clinical therapy.

## Abbreviations

CDFI: Color Doppler flow imaging; CT: Computed tomography; HE: Hematoxylin and eosin stain; NHLL: Non-Hodgkin's lymphoma.

## Competing interests

The authors declare that they have no competing interests.

## Authors’ contributions

All authors read and approved the final manuscript.
